# Definitions of Histological Abnormalities in Inflammatory Bowel Disease: an ECCO Position Paper

**DOI:** 10.1093/ecco-jcc/jjad142

**Published:** 2023-08-22

**Authors:** Roger Feakins, Paula Borralho Nunes, Ann Driessen, Ilyssa O Gordon, Nina Zidar, Pamela Baldin, Britt Christensen, Silvio Danese, Naoimh Herlihy, Marietta Iacucci, Maurice B Loughrey, Fernando Magro, Aart Mookhoek, Magali Svrcek, Francesca Rosini

**Affiliations:** Department of Cellular Pathology, Royal Free London NHS Foundation Trust; University College London; London, UK; Department of Pathology, Hospital Cuf Descobertas, Lisboa and Faculdade de Medicina da Universidade de Lisboa, Lisbon, Portugal; Department of Pathology, University Hospital Antwerp, University of Antwerp, Edegem, Belgium; Department of Pathology, Robert J. Tomsich Pathology and Laboratory Medicine Institute, Cleveland Clinic, Cleveland, OH, USA; Institute of Pathology, Faculty of Medicine, University of Ljubljana, Ljubljana, Slovenia; Department of Pathology, Cliniques Universitaires St-Luc, Université Catholique de Louvain, Brussels, Belgium; Royal Melbourne Hospital Melbourne, Department of Gastroenterology, Parkville; University of Melbourne, Department of Medicine, Melbourne, Victoria, Australia; IRCCS Ospedale and University Vita-Salute San Raffaele, Department of Gastroenterology, Milan, Italy; Department of Cellular Pathology, University College London Hospital NHS Foundation Trust, London, UK; APC Microbiome Ireland, College of Medicine and Health, University College Cork, Cork, Ireland; Patrick G Johnston Centre for Cancer Research, Queen’s University Belfast; Department of Cellular Pathology, Royal Victoria Hospital, Belfast Health and Social Care Trust; Belfast, UK; CINTESIS@RISE, Faculty of Medicine of the University of Porto, Porto, Portugal; Institute of Tissue Medicine and Pathology, University of Bern, Bern, Switzerland; Sorbonne Université, AP-HP, Hôpital Saint-Antoine, Department of Pathology, Paris, France; Pathology Unit, IRCCS Azienda Ospedaliero-Universitaria di Bologna, Bologna, Italy

**Keywords:** Inflammatory bowel disease, histology, definition, ulcerative colitis, Crohn’s disease

## Abstract

Histological assessment of endoscopic biopsies in inflammatory bowel disease [IBD] plays an important role in clinical management, investigative studies, and clinical trials. Scoring schemes consisting of multiple histological items and offering considerable precision are widely available. However, definitions of histological abnormalities are often inconsistent. Furthermore, interobserver variability for their recognition and assessment may be high.

The European Crohn’s and Colitis Organisation [ECCO] formed an expert panel to explore definitions of histological abnormalities in IBD, with the aim of improving the quality of diagnosis and facilitating development of scoring schemes. The process confirmed that the current definitions often have no evidence base and vary between sources. Using available evidence and expert knowledge, the panel produced a series of ECCO consensus position statements on histological features in IBD.

## 1. Introduction

Microscopic examination and interpretation of ileocolonic biopsies help to optimise the management of inflammatory bowel disease [IBD]. Histology can confirm the diagnosis, aid subclassification, determine severity, estimate the efficacy of drug therapy, and detect complications.^1,2^ The pathologist’s focus depends on the setting. For example, basal plasmacytosis and architectural changes support a diagnosis of IBD, and granulomas and spatial distribution help to distinguish between ulcerative colitis [UC] and Crohn’s disease [CD].^1^ Clinical trials and observational studies may require assessment of histological activity and other histological changes,^3–5^ sometimes relying on precise multi-component scoring systems.^6–11^ However, there is often no consensus on basic definitions of histological abnormalities,^9^ and many definitions are inevitably arbitrary. Table 1 outlines broad categories of histological abnormality.

**Table 1:** Broad categories of histological abnormality.

Type of abnormality	Example
Unequivocally abnormal feature	Crypt abscess Granuloma
Features whose occurrence in normal mucosa is a subject of controversy	Lamina propria neutrophils Eosinophil cryptitis
Increase in a feature that is usually absent or sparse	Crypt branching
Increase or reduction in a feature that is normally present	Plasma cells in the basal mucosa [increase] Lymphoid aggregates [increase] Epithelial mucin [reduction]
The severity or magnitude of an abnormality	Mild, moderate, or severe increase in eosinophils / neutrophils / plasma cells
Presence of a feature that is normal at one site but would be abnormal at another site	Paneth cells in right colon [normal] Minor crypt distortion in caecum and rectum [normal]

## 2. Materials and Methods

The project leaders assembled a panel of 15 expert histopathologists and gastroenterologists to develop consensus position statements on histological definitions in IBD. Table 2 summarises the aims. The ECCO Positions appear below with accompanying supporting text. Details of the methodology and additional text are in the Supplementary Materials.

**Table 2.** Aims of the consensus process.

Produce ECCO Positions on histological features in inflammatory bowel disease [IBD]
Reduce interobserver and intra-observer variability
Improve the quality and precision of histological diagnosis of IBD
Refine the assessment of histological activity and severity in IBD
Assist development of new IBD histological scoring schemes

## 3. ECCO Positions

### 3.1. Architecture

**ECCO Position 1.1.**

**Crypt distortion in colorectal mucosa is defined by loss of parallel crypt architecture, an increase in crypt branching, and/or variation in crypt size and shape**

Agreement: 100%

**ECCO Position 1.2.**

**An occasional branched crypt is a normal finding in colorectal mucosa**

Agreement: 93%

**ECCO Position 1.3.**

**Crypt atrophy is a reduction in the proportion of the total colorectal mucosal area that crypts occupy**

Agreement: 87%

**ECCO Position 1.4.**

**Crypt atrophy is recognised by an increase in the distance between crypts, resulting in a distance of more than one crypt diameter between crypts, and/or a general increase in the distance between muscularis mucosae and crypts in well-oriented sections of an adequately sized biopsy**

Agreement: 87%

**ECCO Position 1.5.**

**In inflammatory bowel disease, crypt atrophy is usually accompanied by crypt distortion**

Agreement: 93%

**ECCO Position 1.6.**

**A villiform/irregular mucosal surface in colorectal mucosa describes an undulating surface due to wide crypt mouths that give the appearance of villous projections**

Agreement: 100%

**Figure 1:**
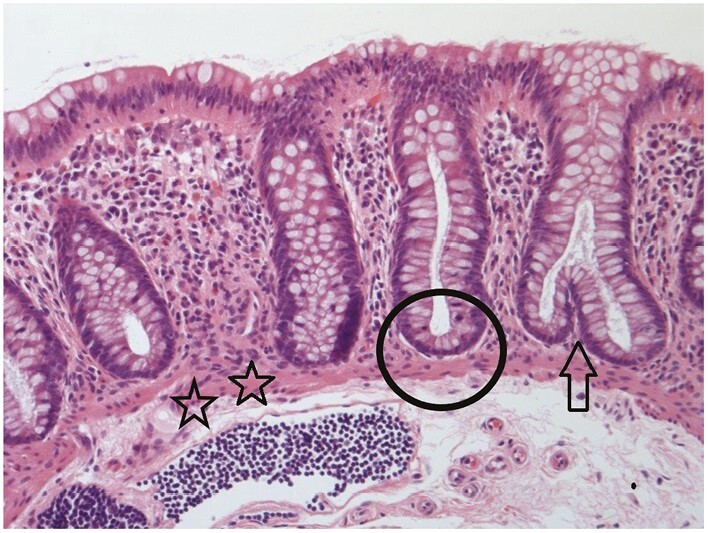
A well-orientated colorectal biopsy, including muscularis mucosae [stars], is ideal for assessment of mucosal architecture. In normal mucosa, crypts are mostly parallel to each other and the crypt base usually reaches the underlying muscularis mucosae [circle]. An occasional branching crypt [arrow] is acceptable as normal. For all colour figures refer to online version.

Normal crypts are parallel to one another and extend from the mucosal surface to the underlying muscularis mucosae [‘test tube’ arrangement; Figure 1].^1,12^ Assessment of architecture and of features that rely on spatial distribution requires biopsies of adequate size with optimal orientation that also ideally include muscularis mucosae [Figure 1].

**Figure 2:**
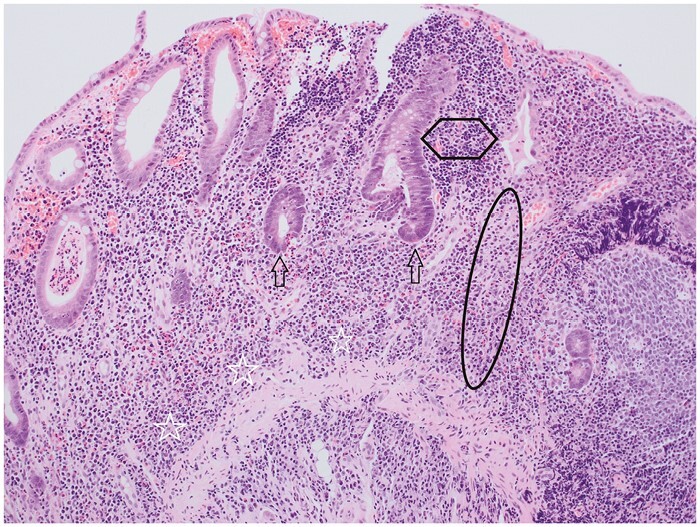
A colonic biopsy that shows extensive crypt distortion, including crypt branching and crypt irregularity. In addition, all crypts show atrophy, with shortening [failure to reach the muscularis mucosae; ellipse] and wider spacing [hexagon]. The two most central crypts show severe epithelial mucin depletion [arrows]. Diffuse basal plasmacytosis is present [stars].

**Figure 3:**
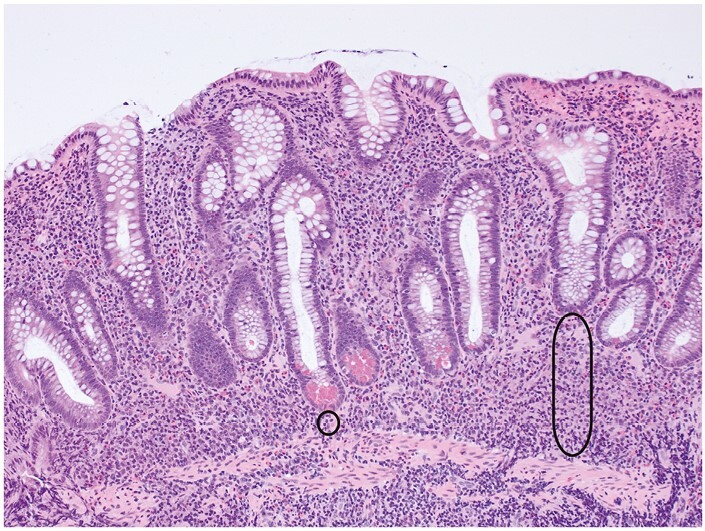
Crypt atrophy and crypt distortion are milder here than in Figure 2. Crypt shortening ranges from very mild [circle] to a reduction of almost 50% in length [capsule]. Mild loss of the normal parallel ‘test tube’ arrangement of crypts represents crypt distortion.

Suggestions for thresholds to define crypt distortion include the following: a minimum of 10% crypts showing any of the features in ECCO Position 1.1. or the presence of more than two branched crypts within a well-oriented biopsy [Figures 1–3].^13–15^ However, there is no consensus on these suggestions.

Crypt atrophy encompasses crypt shortening and wider crypt spacing [Figures 2 and 3], but assessment of these features is subjective.^13,16,17^ Occasional definitions exist [eg, at least one crypt diameter between two crypts].^13,15–21^

Several exceptions are worth noting. Sampling from innominate grooves can demonstrate apparent branching.^22^ The mucosa up to a distance of three crypts from a lymphoid aggregate is probably unreliable for assessment of architecture because the aggregates can distort adjacent glands. Normal rectal or caecal mucosa may show a minor degree of crypt distortion or shortening [Figure 1].^1^

**Figure 4:**
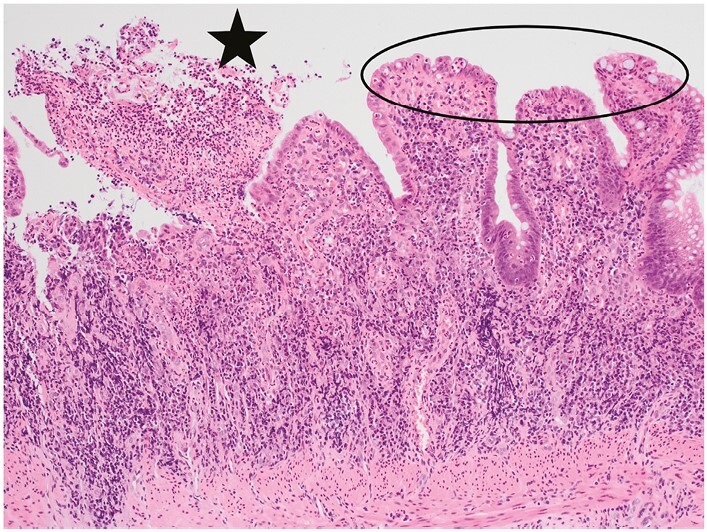
A villiform surface in colorectal mucosa [ellipse] is the consequence of wide crypt mouths and irregular regeneration. At the top left there is an erosion [star], defined as injury to the surface epithelium and underlying mucosa without extension deeper than the muscularis mucosae.

There is no consistent definition of surface irregularity [or the analogous terms villous surface, villiform surface, villous mucosa]. The impression of a villiform surface is partly the result of separation of crypts [Figure 4].^21,23,24^

**ECCO Position 1.7.**

**Villous atrophy in the ileal mucosa is defined as the presence of shortened villi with a villus:crypt ratio lower than 3:1**

Agreement: 93%

### 3.2. Epithelium

**ECCO Position 2.1.**

**Mucin depletion is defined as an unequivocal reduction of goblet cell mucin in crypt or surface epithelium**

Agreement: 93%

**ECCO Position 2.2.**

**Paneth cell metaplasia is defined as the presence of Paneth cells distal to the splenic flexure**

Agreement: 100%

**ECCO Position 2.3.**

**Pyloric metaplasia [synonym pseudopyloric metaplasia] is the replacement of the original epithelium with glands resembling those in the gastric antrum**

Agreement: 93%

Definitions of mucin depletion may specify a reduction in the number of goblet cells or a reduction in mucin droplets [Figure 2].^5,13^ Definitions describe ‘significant’ or ‘unequivocal’ reduction but do not usually offer quantification. One suggestion is one or less goblet cell in eight to ten enterocytes [anonymous personal communication].

**Figure 5:**
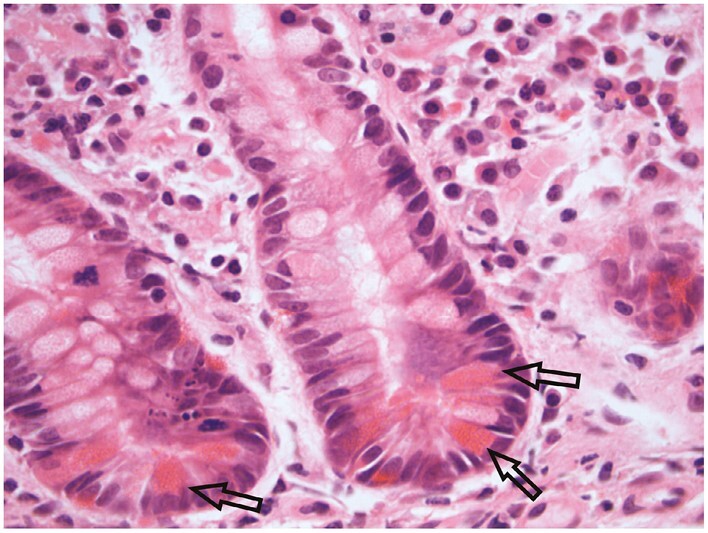
Paneth cells [arrows] with characteristic bright red cytoplasm are numerous at crypt bases in the small bowel and are normal in the right colon. By convention, their presence distal to the splenic flexure constitutes Paneth cell metaplasia.

Paneth cells have a distinctive pyramidal shape and supranuclear eosinophilic granular cytoplasm [Figure 5].^25–28^ Most sources state that Paneth cells distal to the splenic flexure are abnormal, despite some evidence that they are present throughout the large bowel [with a decrease in number from proximal to distal].^28–30^

**Figure 6:**
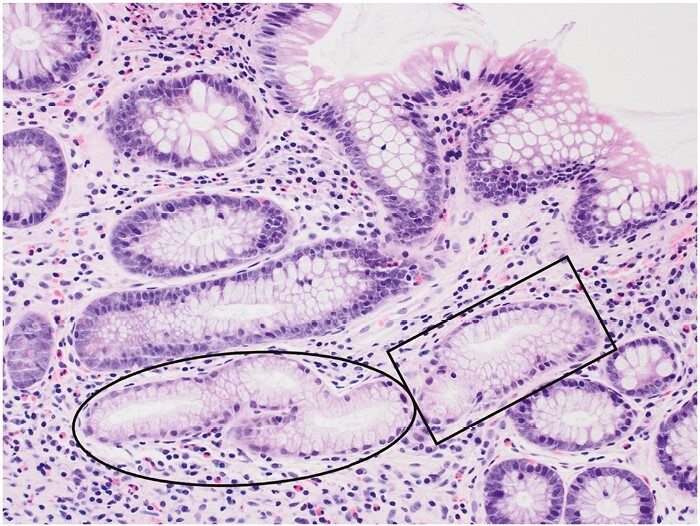
Pyloric metaplasia in ileal mucosa, comprising two glands [ellipse and rectangle] lined by columnar cells with small basal nuclei and pale or clear cytoplasm and resembling gastric pyloric or duodenal Brunner’s glands.

Pyloric metaplasia [PM; broadly synonymous with pseudopyloric metaplasia / ulcer-associated cell lineage / spasmolytic polypeptide-expressing metaplasia] comprises glands lined by columnar cells that have clear or pale PAS-positive cytoplasm with neutral mucin granules and oval or round basal nuclei, thus resembling normal gastric antral and Brunner glands [Figure 6].^31,32^ PM is associated with chronicity, particularly in the small bowel in CD. PM occurs in areas of ulceration and regeneration and may persist long after completion of healing.^33–36^

### 3.3. Chronic Inflammation

**ECCO Position 3.1.**

**In inflammatory bowel disease biopsies, chronic inflammation is defined as an increase in the number of lymphocytes and plasma cells in the lamina propria**

Agreement: 100%

**ECCO Position 3.2.**

**Basal plasmacytosis is defined as an increase in plasma cells at the basal aspect of the colorectal mucosa, typically between the base of the crypts and the muscularis mucosae**

Agreement: 100%

**ECCO Position 3.3.**

**The criteria and thresholds for grading chronic inflammation as absent, mild, moderate, or severe are defined vaguely or are not defined at all**

Agreement: 100%

**Figure 7:**
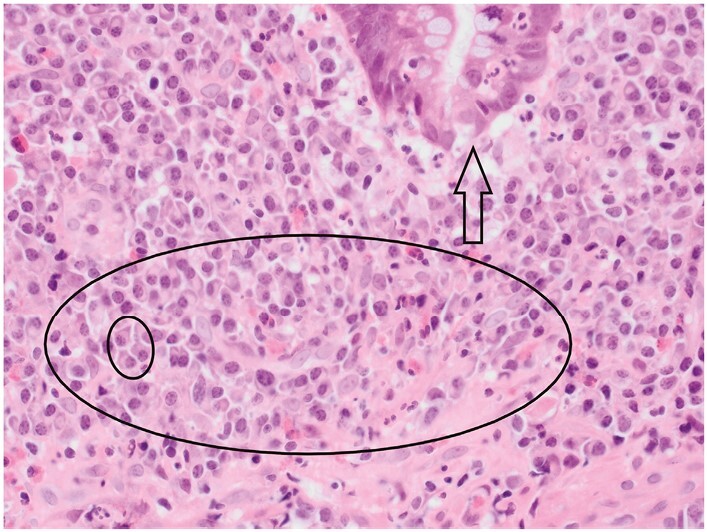
Basal plasmacytosis, comprising an increase in plasma cell numbers at the mucosa base [‘crypts with their feet in pools of plasma cells’]^40^ and elsewhere [eg, within large ellipse; small ellipse surrounds three plasma cells]. Other inflammatory cells [eg, eosinophils] are also apparent. The base of a crypt [arrow] shows cryptitis [ie, at least one neutrophil in the crypt epithelium].

Histological manifestations of mucosal chronic inflammation include basal plasmacytosis, lymphoid aggregates, and a diffuse or variable increase in lamina propria chronic inflammatory cells. Basal plasmacytosis is the most objective of these, is the earliest sign of *de novo* IBD, is the strongest predictor of an IBD diagnosis, and can resolve fully or persist.^37–40^ When basal plasmacytosis is present, the density of plasma cells at the base of the mucosa is high and may be similar to or higher than the density of plasma cells in the upper mucosa [Figures 2, 7, and 9].^1,13,20^ Basal plasmacytosis can be diffuse and band-like, focal, or patchy.^1,41,42^Basal plasmacytosis is more difficult to recognise in the small bowel mucosa than in the colorectal mucosa because there is no plasma cell gradient in normal small bowel mucosa.^43–45^ There is no agreement on how to measure the number of mucosal chronic inflammatory cells or on grading of basal plasmacytosis.^40,43,46^ Although IBD is a chronic inflammatory process,^47,48^ there is less emphasis in clinical practice and in scoring systems on chronic inflammation than on acute or active inflammation.^6^

Some authors refer to basal lymphoplasmacytosis, for which definitions are even less precise than for basal plasmacytosis. Determining colorectal lamina propria lymphocyte numbers or density or defining an increase in lymphocytes is more difficult and subjective than assessing plasma cells.

**ECCO Position 3.4.**

**Basal lymphoid aggregates are nodular collections of lymphocytes in the mucosa. They may disrupt the muscularis mucosae and extend into the superficial submucosa. They may contain germinal centres**

Agreement: 93%

**Figure 8:**
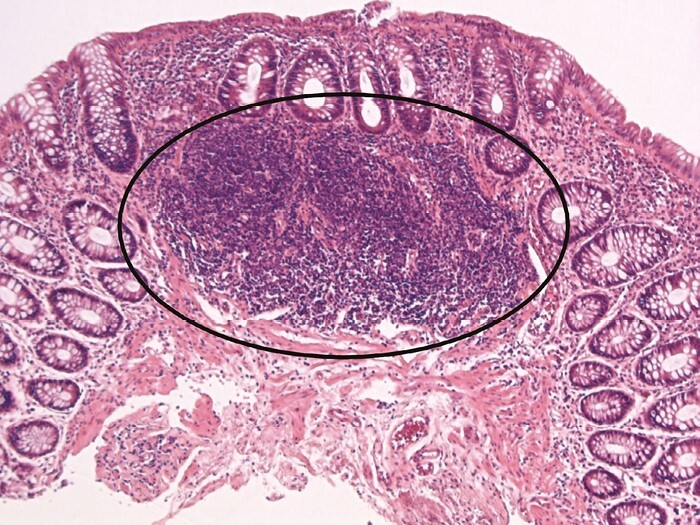
Lymphoid aggregates [ellipse around an aggregate] are nodular collections of lymphocytes that typically lie at the base of the mucosa. They are a normal finding in intestinal mucosa. Their number and density may increase in inflammatory bowel disease [IBD], but defining a significant increase is difficult.

According to some sources, up to two basal lymphoid aggregates may be present in a normal colorectal mucosal biopsy [Figure 8]. However, distinction between normal and abnormal numbers is difficult. Scoring schemes that include lymphoid aggregates do not quantify these aggregates.^49,50^

**ECCO Position 3.5.**

**In inflammatory bowel disease, architectural distortion and epithelial metaplasia are part of the chronic inflammatory process**

Agreement: 100%

Architectural distortion and epithelial metaplasia are the result of crypt destruction and injury associated with chronic and active inflammation, but they do not define ‘chronic inflammation’ in IBD.^51^

### 3.4. Eosinophils

**ECCO Position 4.1.**

**Eosinophils do not define chronic inflammation or acute inflammation reliably in inflammatory bowel disease**

Agreement: 93%

**ECCO Position 4.2.**

**There is no widely accepted definition of a significant increase in colorectal mucosal eosinophils in inflammatory bowel disease**

Agreement: 93%

**ECCO Position 4.3.**

**Eosinophilic cryptitis is defined as the presence of at least one eosinophil in the crypt epithelium**

Agreement: 93%

**ECCO Position 4.4.**

**An eosinophilic crypt abscess is defined as eosinophils in a crypt lumen without the presence of luminal neutrophils**

Agreement: 100%

**Figure 9:**
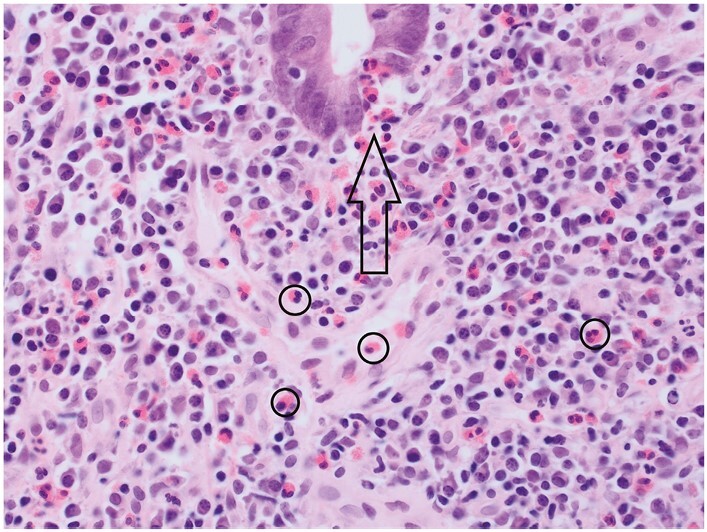
Eosinophils accompany basal plasma cells in this biopsy [circles identify four eosinophils]. A crypt shows eosinophil cryptitis [arrow; ie, at least one eosinophil in the crypt epithelium without accompanying neutrophils]. The maximum number of lamina propria eosinophils and of foci of eosinophilic cryptitis in normal mucosa is uncertain.

**Figure 10:**
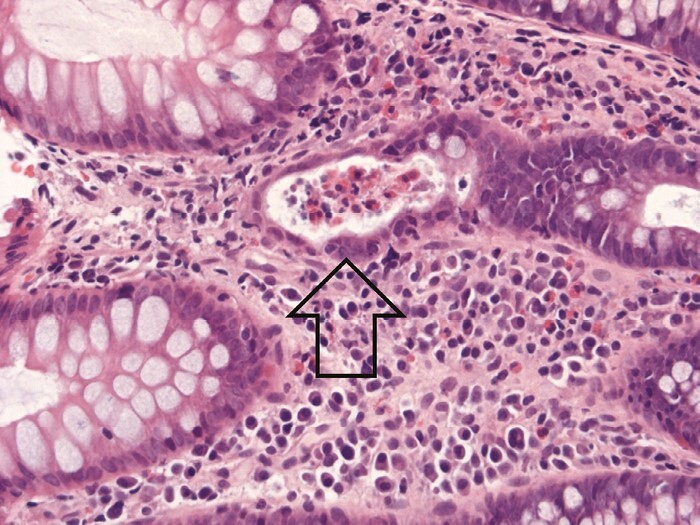
This crypt abscess [arrow] consists mainly of eosinophils but also includes neutrophils. Therefore, it is a crypt abscess rather than an eosinophil crypt abscess.

There is limited information about the normal number of intestinal mucosal eosinophils, the definition of an eosinophil crypt abscess,^50,49^ and the criteria for a mild, moderate, or severe increase in intestinal mucosal eosinophils [Figures 7, 9]. The significance of focal eosinophil cryptitis in the absence of other changes is also uncertain. The panel agreed that an eosinophilic crypt abscess comprises at least two eosinophils. If neutrophils are also present, the term for the lesion is a crypt abscess [ie, neutrophil crypt abscess; Figure 10].

### 3.5. Granulomas

**ECCO Position 5.1.**

**In inflammatory bowel disease, a granuloma is a distinct collection of at least five histiocytes/macrophages that can also contain multinucleate giant cells. It may have a well-defined or poorly defined outline, lacks necrosis, and may have a lymphoid cuff**

Agreement: 93%

**ECCO Position 5.2.**

**A cryptolytic granuloma [mucin granuloma] is a collection of histiocytes, neutrophils, and mucin associated with an injured crypt and can be present in any form of inflammatory bowel disease**

Agreement: 100%

**Figure 11:**
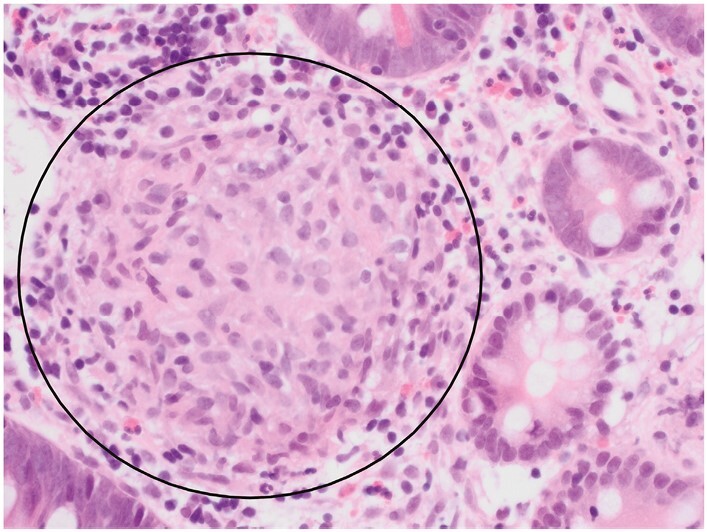
A granuloma [circle] in Crohn’s disease [CD] includes at least five histiocytes and does not show necrosis. Here, the number of histiocytes is considerably greater than five. Multinucleate giant cells and a lymphoid cuff are present in some granulomas in CD, but not in this example.

The number of histiocytes necessary to define a granuloma in IBD is at least five.^13,44,52^ The threshold may vary with the setting.^15^ Histiocyte counts may depend partly on section thickness, number of serial sections examined, and the individual observer. In CD, a granuloma is almost always non-necrotising, never has caseous necrosis, and typically shows no confluence with other granulomas [Figure 11].^44,45,51,53,54^ Giant cells are acceptable. Conventional practice classifies granulomas as either present or absent, with no quantification.

**Figure 12:**
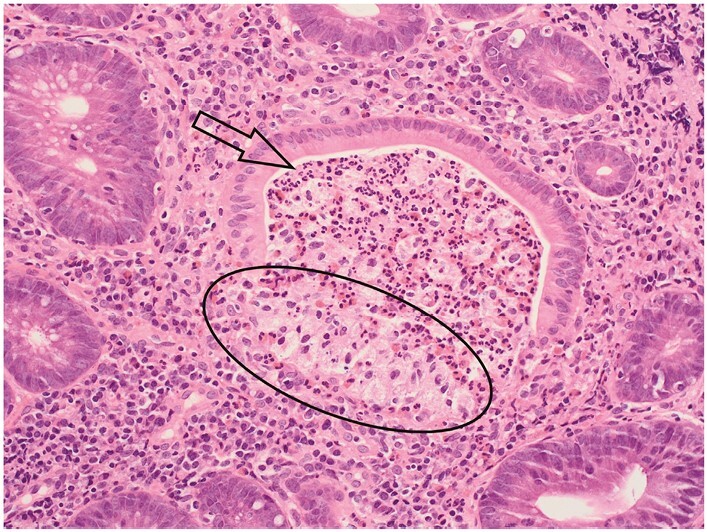
A crypt abscess [arrow] comprises at least two neutrophils in the crypt lumen and may include other inflammatory cells. The deep part of this crypt shows rupture with extrusion of contents, generating a cryptolytic granuloma [circle].

A cryptolytic granuloma characteristically lies adjacent to a crypt that exhibits rupture [Figure 12].^55,56^ In addition to mucin, neutrophils, and histiocytes, the lesion may contain lymphocytes, multinucleate foreign body-type giant cells, and parts of epithelial cells.^44,57^ It may consist largely of histiocytes. Close approximation of a granuloma to a crypt suggests that it is cryptolytic, but identification of crypt injury may require serial tissue sections and is sometimes not possible.^51^

Although granulomas help distinguish CD from UC, most biopsy samples from CD do not contain granulomas.^58^ Absence of a demonstrable association with crypt injury or with ulceration strengthens the discriminatory value of a granuloma towards CD. Cryptolytic granulomas can occur in both CD and UC,^1^ but appear to be more common in CD.

The definition of microgranuloma varies in the literature and there is no widely accepted definition.

### 3.6. Acute inflammation

**ECCO Position 6.1.**

**Acute inflammation is defined histologically by the presence of acute inflammatory cells, namely, neutrophils, in the lamina propria and/or within the surface epithelium, the crypt epithelium, or the lumens of crypts. Acute inflammation can be graded using a validated scoring scheme**

Agreement: 93%

**ECCO Position 6.2.**

**Cryptitis is defined as the presence of at least one neutrophil in the crypt epithelium**

Agreement 100%

**ECCO Position 6.3.**

**A crypt abscess is defined as the presence of neutrophils in the crypt lumen**

Agreement 93%

Most definitions state that the lamina propria and epithelium of normal colorectal mucosa do not contain neutrophils. However, some authors assert that one, two, or even three neutrophils are acceptable, particularly in the lamina propria. There is lack of agreement.^22,24^ Bowel preparation probably accounts for some instances of small numbers of neutrophils. Neutrophils in the capillary lumen in isolation do not indicate acute inflammation.

The panel agreed that one neutrophil is sufficient to define cryptitis, although some publications specify more than one. At least one neutrophil must be demonstrably intraepithelial [Figure 7]. Similarly, there is no universal agreement on the minimum number of neutrophils in a crypt abscess.^15^ Definitions of a crypt abscess may refer to a cluster or chain of neutrophils. General definitions of ‘abscess’ usually require more than one neutrophil [Figures 10, 12]. The panel agreed that the presence of at least two neutrophils is necessary. By convention, the terms ‘cryptitis’ and ‘crypt abscess’ without further qualification refer to neutrophil cryptitis and neutrophil crypt abscesses, respectively. Cryptitis or a crypt abscess can include other types of inflammatory cells in addition to the neutrophil[s] [Figures 10, 12].

Grading of acute inflammation may refer to none, mild, moderate, and severe,^59^ although this is often subjective. Many histology scoring schemes include grading of acute inflammation despite the lack of concordance on methodology. Examples of mild acute inflammation in scoring schemes include the following: few/rare neutrophils in the lamina propria and/or epithelium [Nancy histological index]; lamina propria neutrophils with no cryptitis or crypt abscesses [modified Riley score]; or <5% crypts and/or surface epithelium infiltrated by neutrophils [Geboes score and Robarts Histopathology Index].^8–11,60^ Furthermore, there is no consensus about whether to score all biopsies, one biopsy per anatomical site, an average, or the biopsy with the most severe changes.^60–62^

**ECCO Position 6.4.**

**An erosion is characterised by injury to the surface epithelium and the underlying mucosa with no extension beyond the muscularis mucosae. An ulcer is characterised by injury that extends beyond the muscularis mucosae. Erosions and ulcers may be difficult to distinguish from one another in a mucosal biopsy**

Agreement 87%

**Figure 13:**
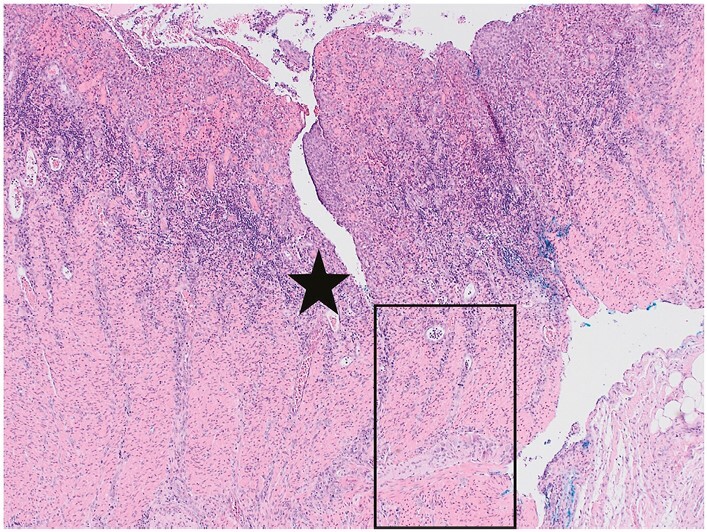
Unlike an erosion, an ulcer extends deep to the muscularis mucosae. In this resection specimen, there is severe ulceration with complete loss of mucosa and with extension into the submucosa [star] and underlying muscularis propria [rectangle]. Distinction from an erosion is easier here than in many mucosal biopsies.

Distinction between an erosion and an ulcer depends on the depth of penetration into the wall.^63^ Erosions and ulcers may be associated with granulation tissue formation, fibrin, a surface exudate that includes neutrophils, vascular proliferation in the lamina propria, and fibroblast proliferation in the submucosa.^63^ Ulcers and erosions may show evidence of re-epithelialisation of the adjacent mucosa. Distinction between erosions and ulcers may be difficult or impossible in practice, especially if biopsies are superficial and lack muscularis mucosae and submucosa [Figures 4, 13].

**3.7. Patterns of injury ECCO Position 7.1.**

**Chronic colitis is a descriptive clinicopathological term that requires histological evidence of chronic mucosal injury. The most consistent and reliable markers of chronic mucosal injury are crypt architectural distortion, basal plasmacytosis, Paneth cell metaplasia, and lamina propria fibrosis**

Agreement 87%

**ECCO Position 7.2.**

**Active chronic colitis is defined as changes that characterise acute inflammation superimposed upon a background of chronic colitis**

Agreement 93%

**ECCO Position 7.3.**

**Chronic ileitis is characterised by a chronic inflammatory cell infiltrate and architectural mucosal distortion, but is often difficult to define and recognise. The presence of pyloric metaplasia in the ileum strongly supports a diagnosis of chronic ileitis**

Agreement 100%

**ECCO Position 7.4.**

**Active chronic ileitis is defined as acute inflammation superimposed upon chronic ileitis**

Agreement 100%

The normal distal ileal mucosa contains confluent dense lymphoid tissue, lymphoid aggregates, and lymphoid follicles. Accordingly, an increase in chronic inflammatory cells alone is usually insufficient to confirm chronic ileitis.^64^ Other features, such as villous distortion, villous atrophy and, in particular, pyloric metaplasia support chronicity in this location.

Clinicians may equate ‘chronic colitis’ with ‘ulcerative colitis’. This term requires qualification, particularly in the conclusion of a pathology report.

Biopsies that show chronicity are categorised as inactive chronic or active chronic, the latter requiring at least one feature of activity. The term ‘activity’ has different meanings depending on the setting [clinical, endoscopy, histology].

**ECCO Position 7.5.**

**Focal active colitis is a histological pattern characterised by cryptitis and/or crypt abscesses involving one or a few crypts, with no associated chronic inflammatory changes**

Agreement 85%

Focal crypt injury by neutrophils and focal active colitis [FAC] are common in endoscopic colorectal biopsies. FAC is associated with epithelial injury. If there is an increase in mononuclear inflammatory cells, the term focal active chronic colitis may be applicable.^65–69^

### 3.8. Distribution of abnormalities

**ECCO Position 8.1.**

**The distribution of the lamina propria inflammatory infiltrate within a single site can be classified as focal, patchy, or diffuse. Focal indicates normal background cellularity with localised areas of increased cellularity; patchy refers to abnormal background cellularity with variable intensity; and diffuse means abnormal background cellularity with an overall increase in density**

Agreement 100%

**ECCO Position 8.2.**

**The distribution of inflammatory changes between different anatomical sites can be classified as continuous or discontinuous. An alternative term for discontinuous is segmental**

Agreement 100%

Assessment of disease distribution macroscopically and microscopically can assist with the distinction between UC and CD.^1,21^

### 3.9. Dysplasia

**ECCO Position 9.1.**

**Dysplasia is an unequivocal neoplastic epithelial alteration without invasive growth**

Agreement 100%

**ECCO Position 9.2.**

**Diagnosis of conventional dysplasia is based on cytological [eg, nuclear hyperchromasia, pseudostratification, enlargement, pleomorphism, and loss of polarity] and architectural [eg, glandular crowding, tubular or villiform architecture, and absence of normal base-to-surface epithelial maturation] alterations of the epithelium**

Agreement 83%

**ECCO Position 9.3.**

**A diagnosis of ‘negative for dysplasia’ is reserved for non-dysplastic epithelium, either normal or regenerative**

Agreement 100%

**ECCO Position 9.4.**

**A standardised classification, the Vienna classification, divides dysplasia into low-grade dysplasia and high-grade dysplasia**

Agreement 93%

**ECCO Position 9.5.**

**Features that favour dysplasia over reactive mucosal changes include diffuse nuclear hyperchromasia, macronucleoli, atypical mitotic figures, loss of cellular polarity, and dirty intraluminal necrosis**

Agreement 100%

**ECCO Position 9.6.**

**Cytological features favouring high-grade dysplasia over low-grade dysplasia include nuclei in the upper half of a cell, complete loss of nuclear polarity, prominent nucleoli, and vesicular chromatin. Architectural changes are also more severe, with cribriform areas**

Agreement 93%

**ECCO Position 9.7.**

**A dysplastic lesion should have at least a few crypts with high-grade changes to be classified as high-grade dysplasia, but the number or proportion required for a final diagnosis of high-grade dysplasia is poorly defined**

Agreement 93%

**ECCO Position 9.8.**

**The category of indefinite for dysplasia is appropriate when a definite distinction between non-neoplastic changes and neoplasia is not possible, regardless of the reasons [eg, poor quality sample, severe inflammatory infiltrate, confusing histological features]**

Agreement 93%

**Figure 14:**
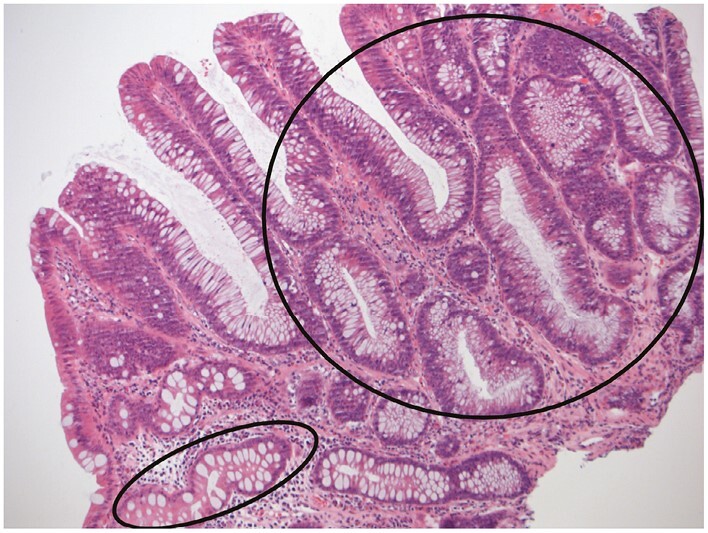
Dysplasia is an unequivocal neoplastic epithelial alteration without invasive growth. The crypt at lower left [ellipse] does not show dysplasia. Most other crypts [eg, within the circle] show low-grade dysplasia, with some resemblance to normal mucosa.

**Figure 15:**
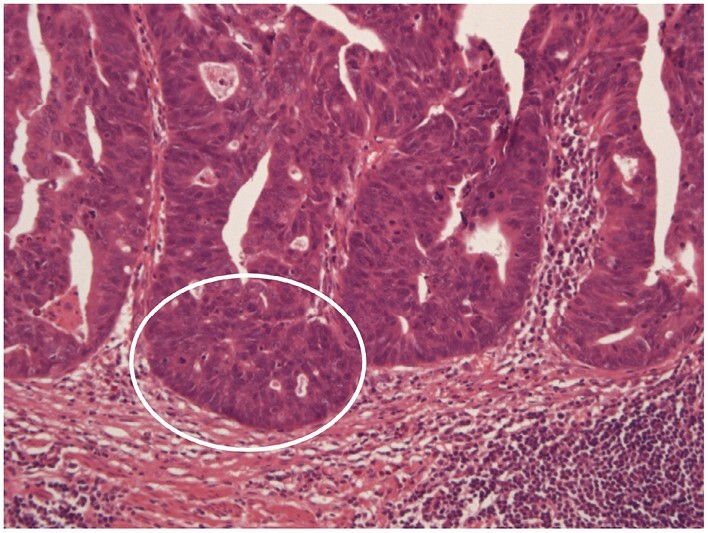
High-grade dysplasia showing severe cytological atypia and severe architectural changes. The latter include a cribriform pattern [ellipse]. The appearances are less reminiscent than low-grade dysplasia of normality.

Assessment of dysplasia in IBD relies on both cytological and architectural features in haematoxylin and eosin [H&E]-stained sections and adheres to the criteria of Riddell *et al*. [Figures 14, 15].^70,71^ The Vienna system and WHO classification propose replacement of the term dysplasia with ‘intraepithelial neoplasia’.^72^ The features of conventional IBD dysplasia are analogous to those characterising gastrointestinal [GI] neoplasia in general [eg, in colorectal adenomas].^14,70,73^ However, the criteria for recognising and grading newer ‘non-conventional’ forms of dysplasia may be different.^74–76^

**Figure 16:**
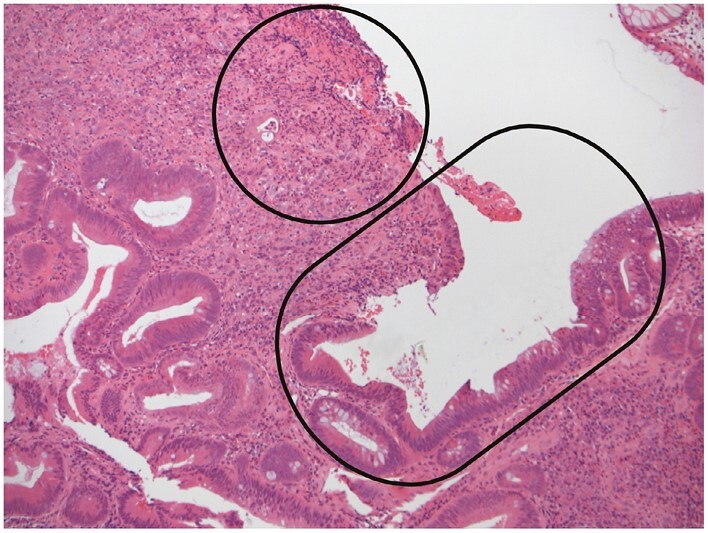
The term ‘indefinite for dysplasia’ is appropriate when distinction between non-neoplastic epithelial changes and dysplasia is not possible, as in this example of epithelial atypia [capsule] adjacent to an ulcer/erosion [circle].

The category ‘indefinite for dysplasia’ includes lesions that are ‘probably negative’ or ‘probably positive’ for dysplasia and is particularly useful when there is significant acute inflammation or ulceration or erosion with epithelial changes that are difficult to classify [Figure 16]. ‘Indefinite for dysplasia’ is not an intermediate category between negative for dysplasia and low-grade dysplasia.

## 4. Discussion

The ECCO Positions in this article represent expert consensus with the support of evidence where available. Table 3 summarises additional considerations. Further development of this process will include attempts to grade histological changes, aiming to improve the consistency of pathological diagnosis and to facilitate development of new scoring schemes for IBD.

**Table 3:** Considerations regarding definitions of histological features of inflammatory bowel disease [IBD].

Abnormality	Present in normal mucosa?	Objective definitions available?	Degree of precision possible	Specificity for IBD	Marker of activity	Reproducibility	References	ECCO Position
Crypt distortion	Sparse/no More likely adjacent to crypt abscesses and lymphoid aggregates or in most distal rectum	Yes, but not precise, eg, ‘two or more branched crypts in a well-oriented biopsy’	Low	++++	No	Variable	^15,18–22,77,78^	1.1, 1.2, 1.5
Crypt atrophy	Rectum and caecum may have wider crypt spacing	Some, eg, ‘more than one crypt diameter between two crypts’	Low	++++	No	Variable	^13,15–17,19–21^	1.3, 1.4, 1.5
Villiform mucosal surface	No	No	Low	++++	No	Variable	^21,40^	1.6
Mucin depletion	May occur near lymphoid follicles or after bowel preparation in normal mucosa	Maybe	Low	++	Maybe	Variable [acceptable for severe depletion]	^16,17,22,79^	
Paneth cell metaplasia	Paneth cells normal in caecum and right colon, and sparse distal to splenic flexure Controversy as to whether distal Paneth cells are normal or not	Yes	Medium	++	No	Variable	^15,22,29^	
Basal plasmacytosis	Basal plasma cells may be normal in the caecum and ascending colon	Subjective Loss of normal plasma cell gradient	Medium	++++	Maybe	Acceptable/good	^19–21,40^	3.2, 7.1
Basal lymphoid aggregates	One or two lymphoid nodules are acceptable in normal mucosa May be between muscularis mucosae and crypts and can extend across the muscularis mucosae	Yes, eg, >2 is abnormal Pathological aggregates difficult to distinguish from normal	Low	++	Maybe	Acceptable	^15,20–22^	3.4
Increase in eosinophil numbers in mucosa	NA	No consensus on definition of an increase No consensus on criteria for mild/moderate/severe increase	Low	+	Maybe	Variable		4.2
Eosinophils cryptitis	Probably, but sparse	Yes Definition of a significant increase uncertain	High	No	Maybe			4.3
Eosinophil crypt abscess	Uncertain; probably very rare	Yes	High	No	Maybe			4.4
Granulomas	No	Yes, eg, ‘a discrete collection of at least five epithelioid macrophages’	High	++	Maybe	Good	^15,17,20^	5.1, 5.2
								2.1
								2.2, 7.1
Neutrophils in lamina propria	No	Yes	High	No	Yes	Variable		
Cryptitis [neutrophil cryptitis]	No	Yes	High	No	Yes	Acceptable	^19,20,77^	6.2
Crypt abscess [neutrophil crypt abscess]	No	Yes	High	no	Yes	Acceptable	^19,20,24,77^	6.3
Ulceration	No	Yes	Medium	No	Yes	Variable		6.4
Erosion	No	Yes	Medium	No	Yes	Variable		6.4

NA, not available.

## Supplementary Material

jjad142_suppl_Supplementary_Material

## Data Availability

No new data were generated in support of this research.
