# Supplementary material for: Definitions of Histological Abnormalities in Inflammatory Bowel Disease: an ECCO Position Paper
Source: J Crohns Colitis. 2023 Aug 22;18(2):175–91. doi: 10.1093/ecco-jcc/jjad142 (PMC10896637; doi:10.1093/ecco-jcc/jjad142)
Supplement: jjad142_suppl_Supplementary_Material [file jjad142_suppl_supplementary_material.docx]

**SUPPLEMENTARY MATERIAL**

METHODS

The project leads [RF and FR] divided the expert panel into four working groups, each of which explored a group of definitions. Broadly, these groups were as follows: architectural and epithelial abnormalities; chronic inflammatory changes; acute inflammatory changes; and terms related to distribution, disease categories, activity, and dysplasia. Sources of evidence included published literature, IBD scoring schemes, practice guidelines, and textbooks. The panel members’ experience and opinion and their familiarity with current practice were also important for the process. Each group developed provisional statements. The threshold for acceptance of a statement was 80% agreement. Two rounds of voting and extensive discussions led to a final set of forty consensus statements.

ADDITIONAL NOTES ON HISTOLOGICAL FEATURES

**Crypts**

The average normal number of crypt profiles corresponding to a 1-mm length of muscularis mucosae is seven to eight. In IBD, there is often a reduction to four to five per mm length or less, such that lamina propria equivalent to or greater than a crypt diameter may separate adjacent crypts.

Crypt atrophy, together with other features [e.g., basal plasmacytosis, abnormal mucosal surface, abnormal crypt architecture], is useful for distinction between IBD and normality but is less effective at distinguishing between ulcerative colitis [UC] and Crohn’s disease [CD].(1) Crypt atrophy is more likely to be present in UC than in CD, possibly because of the heterogeneous distribution of CD.(2)

**Villous surface**

Seldenrijk *et al* proposed a distinction between ‘irregular surface’ and ‘villous surface’ according to the villous:crypt ratio.(3) The ratio is > 1.5 in ‘villous surface’ and is between 1 and 1.5 in ‘irregular surface’. However, this difference is probably not important from a diagnostic perspective.(4-6)

**Villous atrophy**

Villous atrophy is common in CD ileitis. The villi are short, irregular in shape, and broad, and may be completely flat. Villous atrophy may also be present in patients with the ileitis of UC [‘backwash ileitis’](7) and in a variety of other disorders.(8-10) Precise determination of the villus:crypt ratio is often difficult in practice because the boundary between villus and crypt is indistinct.

**Paneth cell metaplasia**

Paneth cells are epithelial cells responsible for secreting antimicrobial alpha defensin peptides and other antimicrobial enzymes that maintain the sterility of intestinal crypts. Paneth cells play a role in defence against microorganisms and in regulation of host immunity.(11) Paneth cells are derived from crypt base columnar cells, which are involved in crypt regeneration and are mostly located in the small intestine but can also be present in the proximal large bowel.(12-15)

Paneth cell metaplasia may reflect repair and regeneration in longstanding disease. Accordingly, Paneth cells are probably a marker of chronicity rather than a discriminant feature.(2, 15, 16) Indeed, Paneth cell metaplasia is not exclusive to IBD and may occur in other colitides [e.g., collagenous colitis and diverticular colitis], but is not a feature of acute self-limiting or infectious colitis.(17) One study reported that Paneth cell metaplasia is more common in UC than in CD, although other studies reported little or no association.(6, 18, 19)

**Pyloric metaplasia**

Pyloric metaplasia [PM] may occur secondary to submucosal responses to ulcer healing. Pathological and molecular descriptions give these metaplastic glands various names that depend partly on the anatomical location, including pyloric metaplasia, pseudopyloric metaplasia, spasmolytic polypeptide-expressing metaplasia [SPEM], and ulcer-associated cell lineage [UACL].(20-24) These reparative metaplasias provide protective mucins and growth factors that probably promote epithelial restitution. Although the cellular origins of the lineages may vary, they all contribute to the resolution of acute and chronic damage to epithelium-lined organs.(20)

The presence of PM in intestinal mucosa suggests chronic disease. PM particularly suggests CD when occurring in persons < 50 years of age without a history of bowel surgery. However, PM also occurs in other chronic inflammatory conditions and is not diagnostic.(25) In terminal ileal CD, PM occurs in 2–27% of ileal endoscopic biopsies and up to 67% of ileal resection specimens.(25-28) Rarely, PM occurs in the ileitis of UC [‘backwash ileitis’](28, 29) and in the colon in CD and UC.(25) Furthermore, PM can be present in the pouch after ileal pouch-anal anastomosis and may predict severe pouch disease.(30-32)

**Eosinophils**

Eosinophil density differs between anatomical segments of the colon (33). Seasonal variation and geographical variation also occur.(33-35) Eosinophils and neutrophils are granulocytes derived from the same cell type, but the physiological role of eosinophils in IBD is more closely related to chronic inflammation and chronic mucosal injury than to acute inflammation.(36, 37) The functional importance of eosinophils in IBD remains obscure(38, 39) and there is limited literature on the clinical significance of eosinophilic cryptitis and other increases in eosinophils in the context of IBD. In a study of UC in paediatric patients, the authors graded eosinophilic infiltration and allocated a higher grade to biopsies that showed eosinophilic cryptitis, eosinophil crypt abscesses, or both.(40)

**Acute inflammation**

Depending on the degree of activity, one or more neutrophils infiltrate the crypt epithelium in cryptitis. In contrast, neutrophils in crypt abscesses are intraluminal and eventually may cause dilatation of the crypt with attenuation of the epithelium. Crypt abscesses are not diagnostic of IBD (41-43). They are generally more numerous and diffuse in UC than in CD.(44) In contrast to a superficial location in infectious colitis, neutrophils in IBD may be more prominent in the basal third of mucosa. However, this is not a reliable discriminatory feature.(3, 46)

**Chronic injury**

The most consistent and reliable markers of chronic colorectal mucosal injury are crypt architectural distortion, basally located lymphoid aggregates, basal plasmacytosis, Paneth cell or pyloric gland metaplasia or hyperplasia in the right colon, and fibrosis of the lamina propria. Chronic colitis can present as chronic crypt-destructive colitis and chronic non-crypt destructive colitis. In non-crypt destructive colitis, evidence of chronicity depends on a mixed, mainly mononuclear inflammatory infiltrate extending into the deep lamina propria without signs of destruction or regeneration and with crypts remaining uniform and parallel.(47, 48) Biopsies demonstrating chronic colitis but preserved crypt architecture may be subclassified as predominantly neutrophilic, predominantly lymphoplasmacytic, or pauci-inflammatory. Regardless of symptom duration at the time of biopsy, signs of regeneration [i.e., branched distorted crypts of varying size, shape, and orientation] and a mononuclear inflammatory infiltrate are present in chronic crypt-destructive colitis.

Inflammation is more likely to be focal and patchy in CD than in UC and more likely to be diffuse in UC than in CD, although there are exceptions.(5, 49, 50)

**Focal active colitis**

Focal crypt injury by neutrophils [cryptitis/crypt abscesses] or focal active colitis [FAC] is a common isolated finding in endoscopic colorectal biopsies.(41, 51-54) Pathologists may fail to notice FAC on low-power examination as the mucosa may have an almost normal appearance.

A minority of patients with FAC either has or develops CD. FAC can also occur in ischaemia, infection, partially treated UC, and as an isolated finding in patients undergoing endoscopy. In one study, the authors classified approximately 20–30% as idiopathic [likely the result of bowel preparation with sodium phosphate].(41, 55, 56) In a paediatric study, 24% of FAC cases had a subsequent diagnosis of IBD. This is higher than in adults, suggesting that FAC is of greater clinical significance in children.(57) In those with FAC, terminal ileal inflammation, crypt abscesses, and elevated serum inflammatory markers were significantly associated with a subsequent diagnosis of IBD. After excluding cases with terminal ileal inflammation, the rate dropped to 11%. This is similar to the rate in adults.(51)

**Inflammatory polyps / pseudopolyps**

Inflammatory polyps [pseudopolyps] are probably areas of residual/remaining mucosa between ulcers during a severe inflammatory process, resulting in mucosal projections in the bowel lumen.(58, 59) They can also develop as a result of the healing and re-epithelialization of previously damaged mucosa. The exact pathogenesis of their formation is not clear. They may present as sessile, pedunculated, or filiform lesions. Histological features range from non-epithelialised granulation tissue to hyperplastic glandular epithelium admixed with inflammatory cells.(58-60) Inflammatory polyps can occur anywhere in the GI tract. The incidence is highest in the large bowel, in particular the transverse colon. They occur more often in UC than CD.(58) Inflammatory polyps are not a marker of IBD activity, can be found in both active colitis and the quiescent phase, and may correlate with disease extent.(58)

**Dysplasia**

Dysplasia in the GI tract is both a precancerous lesion and a marker for a high risk of cancer.(5, 61) Classification of IBD dysplasia as low-grade [LGD] and high-grade dysplasia [HGD](62, 63) results in better interobserver and intraobserver agreement than classification into three groups.(64-66) An international panel of pathologists developed the Vienna grading system as a response to discrepancies between US, European, and Japanese pathologists. This system lists the following five diagnostic categories: (i) negative for neoplasia; (ii) indefinite for neoplasia; (iii) non-invasive low-grade neoplasia; (iv) non-invasive high-grade neoplasia [including non-invasive carcinoma in situ and suspicion of invasive carcinoma]; and (v) invasive neoplasia [including intramucosal carcinoma and submucosal carcinoma or beyond].(63)

The microscopic features allowing a diagnosis of dysplasia are both architectural and cytological. Abnormal growth patterns indicate faulty control of cellular proliferation and include glandular crowding, tubular or villiform architecture, and the absence of normal base-to-surface epithelial maturation. Cytological alterations include nuclear hyperchromasia, pseudostratification, enlargement, pleomorphism, and loss of polarity.

Typically, dysplastic epithelium extends uniformly along the crypt axis and surface epithelium, with little or no surface maturation.(67, 68) However, the nuclei in crypt dysplasia, a rare entity, do not extend to the surface.

The diagnosis of dysplasia can be challenging, especially when there is a background of active or resolving inflammation causing reactive changes. The term ‘indefinite for dysplasia’ is appropriate when definite distinction between non-neoplastic changes and neoplasia is not possible because of factors such as severe inflammation and regeneration, ulceration, or technical artifacts [e.g., too small, too superficial, malorientated, fragmented, poorly fixed, suboptimally stained, presence of marked cautery artifact].(62, 63)

Most studies show significant inter- and intra-observer variability, especially for LGD versus indefinite for dysplasia.(61) Therefore, confirmation of dysplasia and its grade by a pathologist with expertise in gastrointestinal pathology is usually recommended.(39, 69, 70)

Most of the literature on IBD-related dysplasia refers to conventional [or intestinal type] dysplasia, the most common form of dysplasia. Conventional dysplasia is defined by histological features identical to those of sporadic adenomas.

Several additional morphological patterns of dysplasia that can occur in IBD are the focus of recent investigations. The collective term for these novel forms is ‘non-conventional dysplasia’. They include at least the following seven subtypes: (i) hypermucinous [hypermucinous villous]; (ii) goblet cell deficient; (iii) crypt cell dysplasia [or dysplasia with terminal epithelial differentiation]; (iv) dysplasia with increased Paneth cell differentiation; (v) sessile serrated lesion-like dysplasia; (vi) TSA-like; and (vii) serrated NOS.(71-73)

REFERENCES

1. Cross SS, Harrison RF. Discriminant histological features in the diagnosis of chronic idiopathic inflammatory bowel disease: analysis of a large dataset by a novel data visualisation technique. J Clin Pathol. 2002;55(1):51-7.

2. Feakins RM. Ulcerative colitis or Crohn's disease? Pitfalls and problems. Histopathology. 2014;64(3):317-35.

3. Seldenrijk CA, Morson BC, Meuwissen SG, Schipper NW, Lindeman J, Meijer CJ. Histopathological evaluation of colonic mucosal biopsy specimens in chronic inflammatory bowel disease: diagnostic implications. Gut. 1991;32(12):1514-20.

4. Schumacher G, Kollberg B, Sandstedt B. A prospective study of first attacks of inflammatory bowel disease and infectious colitis. Histologic course during the 1st year after presentation. Scand J Gastroenterol. 1994;29(4):318-32.

5. Feakins RM. Inflammatory bowel disease biopsies: updated British Society of Gastroenterology reporting guidelines. J Clin Pathol. 2013;66(12):1005-26.

6. Theodossi A, Spiegelhalter DJ, Jass J, Firth J, Dixon M, Leader M, et al. Observer variation and discriminatory value of biopsy features in inflammatory bowel disease. Gut. 1994;35(7):961-8.

7. Haskell H, Andrews CW, Jr., Reddy SI, Dendrinos K, Farraye FA, Stucchi AF, et al. Pathologic features and clinical significance of "backwash" ileitis in ulcerative colitis. Am J Surg Pathol. 2005;29(11):1472-81.

8. Pallav K, Leffler DA, Tariq S, Kabbani T, Hansen J, Peer A, et al. Noncoeliac enteropathy: the differential diagnosis of villous atrophy in contemporary clinical practice. Aliment Pharmacol Ther. 2012;35(3):380-90.

9. Schiepatti A, Cincotta M, Biagi F, Sanders DS. Enteropathies with villous atrophy but negative coeliac serology in adults: current issues. BMJ Open Gastroenterol. 2021;8(1).

10. Dilauro S, Crum-Cianflone NF. Ileitis: when it is not Crohn's disease. Curr Gastroenterol Rep. 2010;12(4):249-58.

11. Bevins CL, Salzman NH. Paneth cells, antimicrobial peptides and maintenance of intestinal homeostasis. Nat Rev Microbiol. 2011;9(5):356-68.

12. Lewin K. The Paneth cell in disease. Gut. 1969;10(10):804-11.

13. Symonds DA. Paneth cell metaplasia in diseases of the colon and rectum. Arch Pathol. 1974;97(6):343-7.

14. Tanaka M, Saito H, Kusumi T, Fukuda S, Shimoyama T, Sasaki Y, et al. Spatial distribution and histogenesis of colorectal Paneth cell metaplasia in idiopathic inflammatory bowel disease. J Gastroenterol Hepatol. 2001;16(12):1353-9.

15. Pezhouh MK, Cheng E, Weinberg AG, Park JY. Significance of Paneth Cells in Histologically Unremarkable Rectal Mucosa. Am J Surg Pathol. 2016;40(7):968-71.

16. Paterson JC, Watson SH. Paneth cell metaplasia in ulcerative colitis. Am J Pathol. 1961;38(2):243-9.

17. Dundas SA, Dutton J, Skipworth P. Reliability of rectal biopsy in distinguishing between chronic inflammatory bowel disease and acute self-limiting colitis. Histopathology. 1997;31(1):60-6.

18. Lennard-Jones JE, Lockhart-Mummery HE, Morson BC. Clinical and pathological differentiation of Crohn's disease and proctocolitis. Gastroenterology. 1968;54(6):1162-70.

19. Simmonds N, Furman M, Karanika E, Phillips A, Bates AW. Paneth cell metaplasia in newly diagnosed inflammatory bowel disease in children. BMC Gastroenterol. 2014;14:93.

20. Goldenring JR. Pyloric metaplasia, pseudopyloric metaplasia, ulcer-associated cell lineage and spasmolytic polypeptide-expressing metaplasia: reparative lineages in the gastrointestinal mucosa. J Pathol. 2018;245(2):132-7.

21. Meditskou S, Grekou A, Toskas A, Papamitsou T, Miliaras D. Pyloric and foveolar type metaplasia are important diagnostic features in Crohn's disease that are frequently missed in routine pathology. Histol Histopathol. 2020;35(6):553-8.

22. Yokoyama I, Kozuka S, Ito K, Kubota K, Yokoyama Y. Gastric gland metaplasia in the small and large intestine. Gut. 1977;18(3):214-8.

23. Modigliani R, Poitras P, Galian A, Messing B, Guyet-Rousset P, Libeskind M, et al. Chronic non-specific ulcerative duodenojejunoileitis: report of four cases. Gut. 1979;20(4):318-28.

24. Liber AF. Aberrant pyloric glands in regional ileitis. AMA Arch Pathol. 1951;51(2):205-12.

25. Tokuyama M, Dhingra S, Polydorides AD. Clinicopathologic features and diagnostic implications of pyloric gland metaplasia in intestinal specimens. Am J Surg Pathol. 2021;45(3):365-73.

26. Geboes K, Ectors N, D'Haens G, Rutgeerts P. Is ileoscopy with biopsy worthwhile in patients presenting with symptoms of inflammatory bowel disease? Am J Gastroenterol. 1998;93(2):201-6.

27. Koukoulis GK, Ke Y, Henley JD, Cummings OW. Detection of pyloric metaplasia may improve the biopsy diagnosis of Crohn's ileitis. Journal of clinical gastroenterology. 2002;34(2):141-3.

28. Goldstein N, Dulai M. Contemporary morphologic definition of backwash ileitis in ulcerative colitis and features that distinguish it from Crohn disease. Am J Clin Pathol. 2006;126(3):365-76.

29. Langner C, Magro F, Driessen A, Ensari A, Mantzaris GJ, Villanacci V, et al. The histopathological approach to inflammatory bowel disease: a practice guide. Virchows Arch. 2014;464(5):511-27.

30. Kariv R, Plesec TP, Gaffney K, Lian L, Fazio VW, Remzi FH, et al. Pyloric gland metaplasia and pouchitis in patients with ileal pouch-anal anastomoses. Aliment Pharmacol Ther. 2010;31(8):862-73.

31. Agarwal S, Stucchi AF, Dendrinos K, Cerda S, O'Brien MJ, Becker JM, et al. Is pyloric gland metaplasia in ileal pouch biopsies a marker for Crohn's disease? Dig Dis Sci. 2013;58(10):2918-25.

32. Li H, Arslan ME, Lee EC, Qualia CM, Mikula MW, Fu Z, et al. Pyloric gland metaplasia: Potential histologic predictor of severe pouch disease including Crohn's disease of the pouch in ulcerative colitis. Pathol Res Pract. 2021;220:153389.

33. Polydorides AD, Banner BF, Hannaway PJ, Yantiss RK. Evaluation of site-specific and seasonal variation in colonic mucosal eosinophils. Hum Pathol. 2008;39(6):832-6.

34. Collins MH, Capocelli K, Yang GY. Eosinophilic Gastrointestinal Disorders Pathology. Front Med (Lausanne). 2017;4:261.

35. Hurrell JM, Genta RM, Melton SD. Histopathologic diagnosis of eosinophilic conditions in the gastrointestinal tract. Adv Anat Pathol. 2011;18(5):335-48.

36. Al-Haddad S, Riddell RH. The role of eosinophils in inflammatory bowel disease. Gut. 2005;54(12):1674.

37. Filippone RT, Sahakian L, Apostolopoulos V, Nurgali K. Eosinophils in Inflammatory Bowel Disease. Inflammatory Bowel Diseases. 2019;25(7):1140-51.

38. Yantiss RK. Eosinophils in the GI tract: how many is too many and what do they mean? Mod Pathol. 2015;28 Suppl 1:S7-21.

39. Magro F, Langner C, Driessen A, Ensari A, Geboes K, Mantzaris GJ, et al. European consensus on the histopathology of inflammatory bowel disease. J Crohns Colitis. 2013;7(10):827-51.

40. Boyle B, Collins MH, Wang Z, Mack D, Griffiths A, Sauer C, et al. Histologic Correlates of Clinical and Endoscopic Severity in Children Newly Diagnosed With Ulcerative Colitis. Am J Surg Pathol. 2017;41(11):1491-8.

41. Greenson JK, Stern RA, Carpenter SL, Barnett JL. The clinical significance of focal active colitis. Hum Pathol. 1997;28(6):729-33.

42. Warren S, Sommers SC. Pathogenesis of ulcerative colitis. Am J Pathol. 1949;25(4):657-79.

43. Kovari B, Bathori A, Friedman MS, Lauwers GY. Histologic Diagnosis of Inflammatory Bowel Diseases. Adv Anat Pathol. 2022;29(1):48-61.

44. Jenkins D, Balsitis M, Gallivan S, Dixon MF, Gilmour HM, Shepherd NA, et al. Guidelines for the initial biopsy diagnosis of suspected chronic idiopathic inflammatory bowel disease. The British Society of Gastroenterology Initiative. J Clin Pathol. 1997;50(2):93-105.

45. Brown SR, Haboubi N, Hampton J, George B, Travis SP. The management of acute severe colitis: ACPGBI position statement. Colorectal Dis. 2008;10 Suppl 3:8-29.

46. Jenkins D, Goodall A, Scott BB. Simple objective criteria for diagnosis of causes of acute diarrhoea on rectal biopsy. J Clin Pathol. 1997;50(7):580-5.

47. Tanaka M, Riddell RH, Saito H, Soma Y, Hidaka H, Kudo H. Morphologic criteria applicable to biopsy specimens for effective distinction of inflammatory bowel disease from other forms of colitis and of Crohn's disease from ulcerative colitis. Scand J Gastroenterol. 1999;34:55-67.

48. Carpenter HA, Talley NJ. The importance of clinicopathological correlation in the diagnosis of inflammatory conditions of the colon: histological patterns with clinical implications. The American Journal of Gastroenterology. 2000;95(4):878-96.

49. Moore M, Feakins RM, Lauwers GY. Non-neoplastic colorectal disease biopsies: evaluation and differential diagnosis. J Clin Pathol. 2020;73(12):783-92.

50. Stange EF, Travis SP, Vermeire S, Reinisch W, Geboes K, Barakauskiene A, et al. European evidence-based Consensus on the diagnosis and management of ulcerative colitis: Definitions and diagnosis. J Crohns Colitis. 2008;2(1):1-23.

51. Osmond A, Ashok D, Francoeur CA, Miller M, Walsh JC. Is focal active colitis of greater clinical significance in pediatric patients? A retrospective review of 68 cases with clinical correlation. Hum Pathol. 2018;74:164-9.

52. Ozdil K, Sahin A, Calhan T, Kahraman R, Nigdelioglu A, Akyuz U, et al. The frequency of microscopic and focal active colitis in patients with irritable bowel syndrome. BMC Gastroenterol. 2011;11:96.

53. Volk EE, Shapiro BD, Easley KA, Goldblum JR. The clinical significance of a biopsy-based diagnosis of focal active colitis: a clinicopathologic study of 31 cases. Mod Pathol. 1998;11(8):789-94.

54. Wightman HR. Active focal colitis. Hum Pathol. 1998;29(8):887-8.

55. Shetty S, Anjarwalla SM, Gupta J, Foy CJ, Shaw IS, et al. Focal active colitis: a prospective study of clinicopathological correlations in 90 patients. Histopathology. 2011;59(5):850-6.

56. Sinagra E, Raimondo D, Pompei G, Fusco G, Rossi F, Tomasello G, et al. Focal active colitis as a predictor of inflammatory bowel disease: results from a single-center experience. J Biol Regul Homeost Agents. 2017;31(4):1119-25.

57. Xin W, Brown PI, Greenson JK. The clinical significance of focal active colitis in pediatric patients. Am J Surg Pathol. 2003;27(8):1134-8.

58. Politis DS, Katsanos KH, Tsianos EV, Christodoulou DK. Pseudopolyps in inflammatory bowel diseases: Have we learned enough? World J Gastroenterol. 2017;23(9):1541-51.

59. Jalan KN, Walker RJ, Sircus W, McManus JP, Prescott RJ, Card WI. Pseudopolyposis in ulcerative colitis. Lancet. 1969;2(7620):555-9.

60. Kelly JK, Gabos S. The pathogenesis of inflammatory polyps. Dis Colon Rectum. 1987;30(4):251-4.

61. Eaden J, Abrams K, McKay H, Denley H, Mayberry J. Inter-observer variation between general and specialist gastrointestinal pathologists when grading dysplasia in ulcerative colitis. J Pathol. 2001;194:152-7.

62. Riddell RH, Goldman H, Ransohoff DF, Appelman HD, Fenoglio CM, Haggitt RC, et al. Dysplasia in inflammatory bowel disease: standardized classification with provisional clinical applications. Hum Pathol. 1983;14:931-68.

63. Schlemper RJ, Riddell RH, Kato Y, Borchard F, Cooper HS, Dawsey SM, et al. The Vienna classification of gastrointestinal epithelial neoplasia. Gut. 2000;47(2):251-5.

64. Public Health England. Bowel cancer screening: pathology guidance on reporting lesions 2021 [Available from: <https://www.gov.uk/government/publications/bowel-cancer-screening-reporting-lesions/bowel-cancer-screening-guidance-on-reporting-lesions>.

65. Talbot I, Price A, Salto-Tellez M. Biopsy pathology in colorectal disease. 2nd ed. London: Hodder Arnold; 2006.

66. Svrcek M. FR. Gastrointestinal Dysplasia. In: Feakins RM, editor. Non-Neoplastic Pathology of the Gastrointestinal Tract: A Practical Guide to Biopsy Diagnosis. Cambridge: Cambridge University Press; 2020. p. 116-30.

67. Patil DT, Odze RD. Biopsy diagnosis of colitis: an algorithmic approach. Virchows Archiv. 2018;472(1):67-80.

68. Patil DT, Odze RD. Inflammatory disorders of the large intestine. In: Odze RD, Goldblum JR, editors. Surgical pathology of the GI tract, liver, biliary tract and pancreas. 4th ed. Philadelphia: Saunders Elsevier; 2023. p. 496-586.

69. Eaden JA, Mayberry JF, British Society for G, Association of Coloproctology for Great B, Ireland. Guidelines for screening and surveillance of asymptomatic colorectal cancer in patients with inflammatory bowel disease. Gut. 2002;51 Suppl 5:V10-2.

70. Lamb CA, Kennedy NA, Raine T, Hendy PA, Smith PJ, Limdi JK, et al. British Society of Gastroenterology consensus guidelines on the management of inflammatory bowel disease in adults. Gut. 2019;68(Suppl 3):s1-s106.

71. Choi WT, Yozu M, Miller GC, Shih AR, Kumarasinghe P, Misdraji J, et al. Nonconventional dysplasia in patients with inflammatory bowel disease and colorectal carcinoma: a multicenter clinicopathologic study. Mod Pathol. 2020;33(5):933-43.

72. Choi WT, Kovari BP, Lauwers GY. The Significance of Flat/Invisible Dysplasia and Nonconventional Dysplastic Subtypes in Inflammatory Bowel Disease: A Review of Their Morphologic, Clinicopathologic, and Molecular Characteristics. Adv Anat Pathol. 2022;29(1):15-24.

73. Akarca FG, Yozu M, Alpert L, Kovari BP, Zhao L, Salomao M, et al. Non-conventional dysplasia is frequently associated with low-grade tubuloglandular and mucinous adenocarcinomas in inflammatory bowel disease. Histopathology. 2023.
